# Supplementary material for: The translation attenuating arginine-rich sequence in the extended signal peptide of the protein-tyrosine phosphatase PTPRJ/DEP1 is conserved in mammals
Source: PLoS One. 2020 Dec 9;15(12):e0240498. doi: 10.1371/journal.pone.0240498 (PMC7725344; doi:10.1371/journal.pone.0240498)
Supplement: S1 Fig — (PDF) [file pone.0240498.s001.pdf]

S1 Fig. Alignment of the 5′ end of the *PTPRJ* transcripts encoding the extended signal peptides in placental mammals.

```
Human            1 ---AGCCCCAGCCGCATGACGCGCGGAGGAGGCAGCGGGA
Mouse            1 -------GGAGCCGCATGACGCGCGGAGGAGGCAGAGGGA
Beluga           1 TGCAGCCCCATCCGCATGACGCGCGGAGGCGGCAGCCGGA
Cattle           1 ---------AGCCGCATGACGCGCGGAGGAGGCAGCGGGA
Otter              ----------------------------------------
Consensus                   AGCCGCATGACGCGCGGAGGAGGCAGCGGGA

Human           38 GCAGCCGCGGG------AGCCGGGACCGGGTAGCCGCGCG
Mouse           34 GCAGCCGGGGCCGCGGGAGCCGGGAGCTGGGAGCCACGCG
Beluga          41 GCCGCCGG---------AGCCGGGATCAGGGCGCTGAGCG
Cattle          32 GCCGCCGGGGCCGCGGGAGCCGGGATCCGGGCGCCGCGCG
Otter              ----------------------------------------
Consensus          GC GCCGGGG       AGCCGGGA C GGG GCCGCGCG

Human           72 CTGGGGGTGGGCGCCGCTCGCTCCGCCCCGCGAAGCCCCT
Mouse           74 CGGAGGGTGGGCGCCGCTCGCTCCGCCCCGCGAAGCCCCT
Beluga          72 CGGGGGGTGGGCGCCGCTCGCTCCGCCCTGCGAAGCCCCT
Cattle          72 CCAGGGGTGGGCGCCGCTTGCTCCGCCCTGCGAAGCCCCT
Otter            1 --------------------------------AAGCCCCT
Consensus          C GGGGGTGGGCGCCGCTCGCTCCGCCC GCGAAGCCCCT

Human          112 GCGCGCTCAGGGACGCGGCCCCCCCGCGGCAGCCGCGCTA
Mouse          114 GCGAGTCTAAGGCCGCGGCCGCTCCGCGCCAGGCGCGCTA
Beluga         112 GCGCGCCCAGGGACGCGTCCACCCCGCTGCAGCCCCGCCA
Cattle         112 AGGCGTCCGGGGACGCGTTCCCCCCGCCGCAGCCGCGCCG
Otter            9 GCGCGCCCAGGGACGCGTCCCTCCCGCGGCAGGCGCGCCA
Consensus          GCGCGCCCAGGGACGCGTCCCCCCCGCGGCAGCCGCGCCA

Human          152 GGCTCCGGCGTGTGGCCGCGGCCGCCGCCGCCGC---TGC
Mouse          154 GGCTCCGCCGTGTG------GCCGCCGCCGCCGCCGCTGC
Beluga         152 GGCTCCGCCGTGTGGCCGCTGCCGCCGCCGCTGC---TGC
Cattle         152 GGCTCCGCCGTGTG------GCCGCCGCCGCCGCCGCTGC
Otter           49 GGCTCCGCCGTGTGGCCGCCGCCGCCGCCGCCGCCGCTGC
Consensus          GGCTCCGCCGTGTGGCCGC GCCGCCGCCGCCGCCGCTGC

Human          189 CATGTCTCCGGGGAAGCCCGGGGCGGGCGGAGCGGGGACG
Mouse          188 CATGTCCCCGGGGAAGCCCGGGGCGGGCGGAGCGGGGACT
Beluga         189 CATGTCCCCGGGGAAGCCCGGGGCGGGCGGAGCGGGGACG
Cattle         186 CATGTCCCCGGGGAAGCCCGGGGCGGGCGGAGCGGGGACG
Otter           89 CATGTCCCCGGGGAAGCCCGGGGCGGGCGGAGCGGGGACT
Consensus          CATGTCCCCGGGGAAGCCCGGGGCGGGCGGAGCGGGGACG

Human          229 AGGCGGACCGGCTGGCGGAGGAGGAGGCGAAGGAGACGGC
Mouse          228 AGGCGGACCGGCTGGCGGAGAAGGAGGCGGAGGCGTCGGC
Beluga         229 AGGCGGACCGGCTGGAGGAGGAGGAGGCGGAGGCGGCGGC
Cattle         226 AGGCGGACCGGCGGGAGGAGGCGGAGGCGGAGGCGGCGGC
Otter          129 AGGCGGACGGGCTGGAGGAGGAGGAGGAGGAGGCGGCGGC
Consensus          AGGCGGACCGGCTGGAGGAGGAGGAGGCGGAGGCGGCGGC

Human          269 AGGAGGCGGCGACGACGGTGCCCGGGCTCGGGCGCACGGC
Mouse          268 TGGAGACGGAGACGAGGGCGCCCGGCTTCGGGCACACGGC
Beluga         269 TGGAGGCGGCGACTAGGGCGCCGGGGCTCGGAAGAACGGT
```

```
Cattle          266 TGGAGGCGGTGACTCAGGCGCCGGGGCTCGGGCGCACGGC
Otter           169 TAGAGGCGGCGACTAGGGCGCCCGGGGTCGGGCGCACGGC
Consensus           TGGAGGCGGCGACTAGGGCGCCCGGGCTCGGGCGCACGGC

Human           309 GGGGCCCGATTCGCGCGTCCGGGGCACGTTCCAGGGCGCG
Mouse           308 GGG---------GCGCGTCCCGGGCACGTTCCAGGGCGCG
Beluga          309 GGGGCCCTACTCGCGCGTCCCGGGCACGTTCCAGGGCGCG
Cattle          306 GGGGCCCGGCTCGCGCGTCCCGGGCACGTTCCAGGGCGCG
Otter           209 GGGGCCCGACTCGCGCGTCCCGGGCACGTTCCAGGGCGCG
Consensus           GGGGCCCGACTCGCGCGTCCCGGGCACGTTCCAGGGCGCG

Human           349 CGGGGCATGAAGCCGGCGGCGCGGGAGGCGCGGCTGCCTC
Mouse           339 CAGGGCATGAAGCCCGCGGCGCGGGAGACGCGGACACCCC
Beluga          349 CGGGGCATGAAGCAGGCGGCGCGGGAGGCGCGGCCGCCTC
Cattle          346 CGGGGCATGAAGCCGGCGGCTCGGGAGGCGCGGCCGCCTC
Otter           249 CGGGGCATGAAGCGGGCGACGCGGGAGGCGCGGCCGCCTC
Consensus           CGGGGCATGAAGCCGGCGGCGCGGGAGGCGCGGCCGCCTC

Human           389 CGCGCTCGCCCGGGCTGCGCTGGGCGCTGCCGCTGCTGCT
Mouse           379 CGCGCTCGCCCGGGCTCCGCTGGGCGCTGCTGCCGCTGCT
Beluga          389 CGCGCTCGCCCCGGCTCCGCTGGGCGCTGCCGCCGCTGCT
Cattle          386 CGCGCTCGCCCGGGCTGCGCTGGGCGCTGCCGCCGCTGCT
Otter           289 CGCGCTCGCCGGGGCTGCGCTGGGCGCTGCCGCCGCTGCT
Consensus           CGCGCTCGCCCGGGCTGCGCTGGGCGCTGCCGCCGCTGCT
                              first exon ▼ second exon
Human           429 GCTGCTGCTGCGCCTGGGCCAGATCCTGTGC------
Mouse           419 GCTGTTGCTACGCCAGGGCCAGGTCCTGTGC------
Beluga          429 GCTGCTGTTGCGCCTGGGCCAGATCTTGTGTACAGGT
Cattle          426 GCTGCTGTTGCGCTTGGGCCAGATCCTGTGC------
Otter           329 GCTGCTGTTGCGCCTGGGCCAGATGGTGTGCGCA---
Consensus           GCTGCTGTTGCGCCTGGGCCAGATCCTGTGC
```

The AUG/ATG triplets (green) upstream of the hydrophobic signal sequence are in-frame with the main protein. The regions encoding the conserved Arg-cluster (yellow) and the hydrophobic region (grey) are marked. The signal peptidase cleavage sites were predicted *in silico*. The end of exon 1 in human *PTPRJ* is marked (▼).
