# Supplementary material for: The translation attenuating arginine-rich sequence in the extended signal peptide of the protein-tyrosine phosphatase PTPRJ/DEP1 is conserved in mammals
Source: PLoS One. 2020 Dec 9;15(12):e0240498. doi: 10.1371/journal.pone.0240498 (PMC7725344; doi:10.1371/journal.pone.0240498)
Supplement: S2 Fig — (PDF) [file pone.0240498.s002.pdf]

S2 Fig. Alignment of the extended signal peptides of PTPRJ in placental mammals.

```
Human             1  MSPGKPGAGGAGTRRTGWRRRRRRRRQEAATTVPGLGRTA
Mouse             1  MSPGKPGAGGAGTRRTGWRRRRRRRRLETETRAPGFGHTA
Beluga            1  MSPGKPGAGGAGTRRTGWRRRRRRRRLEAATRAPGLGRTV
Cattle            1  MSPGKPGAGGAGTRRTGGRRRRRRRRLEAVTQAPGLGRTA
Otter             1  MSPGKPGAGGAGTRRTGWRRRRRRRRLEAATRAPGVGRTA
Consensus            MSPGKPGAGGAGTRRTGWRRRRRRRRLEAATRAPGLGRTA

Human            41  GPDSRVRGTFQGARGMKPAAREARLPPRSPGLRWALPLLL
Mouse            41  G---RVPGTFQGAQGMKPAARETRTPPRSPGLRWALLPLL
Beluga           41  GPYSRVPGTFQGARGMKQAAREARPPPRSPRLRWALPPLL
Cattle           41  GPGSRVPGTFQGARGMKPAAREARPPPRSPGLRWALPPLL
Otter            41  GPDSRVPGTFQGARGMKRATREARPPPRSPGLRWALPPLL
Consensus            GP SRVPGTFQGARGMKPAAREARPPPRSPGLRWALPPLL

Human            81  LLLRLGQILC▼AG
Mouse            78  LLLRQGQVLC▼AG
Beluga           81  LLLRLGQILCTG▼DC
Cattle           81  LLLRLGQILC▼AD
Otter            81  LLLRLGQMVCA▼GD
Consensus            LLLRLGQILC
```

Translation starts at the second AUG triplet in the mRNA (see S1 Fig.). The initiating Met residues (green), the conserved Arg-clusters (yellow), the hydrophobic region (grey), and the cleavage sites of the signal peptidase (▼) are shown.
