# Supplementary material for: The translation attenuating arginine-rich sequence in the extended signal peptide of the protein-tyrosine phosphatase PTPRJ/DEP1 is conserved in mammals
Source: PLoS One. 2020 Dec 9;15(12):e0240498. doi: 10.1371/journal.pone.0240498 (PMC7725344; doi:10.1371/journal.pone.0240498)
Supplement: S3 Fig — (PDF) [file pone.0240498.s003.pdf]

S3 Fig. Alignment of the 5′ end of the *PTPRJ* transcripts encoding the extended signal peptides in marsupials.

```
Opossum          1 AGGGAGGGGGCTGGCTTCTCCCGGAGGTGGCGGCTGC---
Koala            1 -GGGAGGGGGTTGGCTTCTCCC---GGCGGCTGCTGC---
T. devil         1 ---GGGGGGGCTGGCTTCTCCCGGCGGCGGCGGCGGCGGC
Wombat             ----------------------------------------
Consensus           ggGaGGGGGcTGGCTTCTCCCgg GG GGCgGCtGC

Opossum         38 AGGGAGCCCGAGCAGCGGGAGCCGCCGGAGCCC---GAG-
Koala           34 AGGGAGCCCGAGCCACGGGAGCCGCCGGAGCCA---CCGG
T. devil        38 AGGGAGCCCGAGCCGCGGGAGCCTCCGGAGCCACCGGAG-
Wombat             ----------------------------------------
Consensus          AGGGAGCCCGAGC gCGGGAGCCgCCGGAGCC    gaG

Opossum         74 -----CCCGAGCCCGAGC----A-CCGG--------AGCC
Koala           71 AGCCGCCGGAACCCGAGC----AGCCGG--------AGCC
T. devil        77 -----CCCGAGCGCGAGCCGGAA-CCGAGCAGTCGCAGCC
Wombat             ----------------------------------------
Consensus               CCcGAgCcCGAGC    A CCGg        AGCC

Opossum         96 CAAGCCGCAGGACGCGTGGAGTAGGCAGCGGGAGCCCGAG
Koala           99 CGAGCCGCAGGACGCGTGGAGCAGGCAGCTGGA-------
T. devil       111 CGAACCACAGGAGGCGTGGAGTCGGCAGCTGGAGCCCGAG
Wombat             ----------------------------------------
Consensus          C AgCCgCAGGAcGCGTGGAGtaGGCAGC GGAgcccgag

Opossum        136 CCGCCCGAGCCGCCCAAGCCGCCCTAGCCTGGACGCCCCC
Koala          132 -----------GCCCAAGCCGCCCGAGCCTGGACGCCGCC
T. devil       151 CCGCCCG-------------------AGCCTG---------
Wombat             ----------------------------------------
Consensus          ccgcccg    gcccaagccgccc AGCCTGgacgcc cc

Opossum        176 GCCCCCGCCGCCGCCGCGCCTCTGCAGCTCGGGGGGGTGG
Koala          161 GCCTCCGCCGCCGCCGCGCCGCTGCAGCTC-GGGGGGTGG
T. devil       164 ------GCCGCCGCCGCGCCGCTGCAGCTCG-GGGGGTGG
Wombat             ----------------------------------------
Consensus          gcc ccGCCGCCGCCGCGCC CTGCAGCTCggGGGGGTGG

Opossum        216 GCGCCGCTTGCTCCGCCCCGTGGAAGCCCTCCTGGCCGCC
Koala          200 GCGCCGCTCGCTCCGCCCCGTGGAAGCCCTCCTGGCCGCC
T. devil       197 GCGCCGCTCGCTCCGCCCCGTGGAAGCCCTCCCGGCCGCC
Wombat             ----------------------------------------
Consensus          GCGCCGCT GCTCCGCCCCGTGGAAGCCCTCCtGGCCGCC

Opossum        256 GCCACCCCCGCCGCCTTCTTCACCGAGGGACTCGGGGCTG
Koala          240 GCCGCCTCCG------------CCGAGGGACGCGGGGCTG
T. devil       237 GCCGCCGCCTCCG---------CCGAGGGACGCGGGGCTG
Wombat             ----------------------------------------
Consensus          GCC CC CCgccg         CCGAGGGAC CGGGGCTG
```

```
Opossum         296 CTCCCGCCCGTTCGAGACTCAGCCTGGTGGTGGCCGCCGC
Koala           268 CTCCCGCCCGCCCGAGACCCAGCCTGGTGGTGGCCGCCGC
T. devil        268 CTCCCGCCCGCCCGAGACCCAGGCTGCCGGTGGCCGCCGC
Wombat              ----------------------------------------
Consensus           CTCCCGCCCG  CGAGAC CAGcCTGgtGGTGGCCGCCGC

Opossum         336 ---TGCCATGTCCCCGGGGAAGCCCGGGGCGGGCGGAGCG
Koala           308 CGCTGCCATGTCCCCGGGGAAGCCCGGGGCGGGCGGAGCG
T. devil        308 CGCTGCCATGTCCCCGGGGAAGCCCGGGGCGGGCGGAGCG
Wombat            1 -------ATGTCCCCGGGGAAGCCCGGGGCGGGCGGAGCG
Consensus              TGCCATGTCCCCGGGGAAGCCCGGGGCGGGCGGAGCG

Opossum         373 GAGAGGAGGCGGAGGAGCTGGAGGCGGCGGCGGCGGCGGC
Koala           348 GAGAGGAGGCGGAGGAGCTGGAGGCGGCGTCGGCGGCGGC
T. devil        348 GAGAAGAGGCGGAGGACGTGGAGGCGGCGGCGGCGGCGGC
Wombat           34 GAGAGGAGGCGGAGGAGCTGGAGGCGGCGGCGGCGGCGGC
Consensus           GAGAGGAGGCGGAGGAGCTGGAGGCGGCGGCGGCGGCGGC

Opossum         413 GGCCTCGGCCCCGGCCACCGGCTCCAGCT-----------
Koala           388 GGCCTCGGCCCCGGCCACCGGCTC----------------
T. devil        388 GGCCTCGGCCCGGGCCCCCGGCTGCGGCTCCCGGAGCCCA
Wombat           74 GGCCTCGGCCCCGGCCACCG------GCT-----------
Consensus           GGCCTCGGCCCCGGCCACCGGCTcc GCT

Opossum         442 -------GCTGTCCCCGGCGCCGAGGCTGCTGCAG---AA
Koala           412 ---------CTGCCCCGGGCGCCGAGGCTGCTGCTG---AA
T. devil        428 GGCTGCTGCGGCTCCCGGAGCCCAGGCTGCTGCCGCTGAA
Wombat           97 -------GCTGCCCCGGGCGCCGAGGCTGCTGCTG---AA
Consensus                  GCTG CCCcGGCGCCGAGGCTGCTGC G   AA

Opossum         472 GCTGCTGCTCTGGGGCCCCAGAGAGCCGCGCTGCTCCCGG
Koala           441 GCTGCTGCTCCGGGGCCCCGGCGCGCCGCGCTGCTCCCGG
T. devil        468 GCCGCCGCTCCGGGGCCCCGCCGCGCCGCGCCGCTCCCGG
Wombat          127 GCTGCTGCTCCGGGGCCCCGGCGCGCCGCGCTGCTCCCGG
Consensus           GCTGCTGCTC GGGGCCCC G G GCCGCGCTGCTCCCGG

Opossum         512 GCACGTTCAGGGACGCTCGGAGCCCGAAGCCCGGGGGGGC
Koala           481 GCTCGTTCCGGGGCGCGCGGAGCCCGAAGCCCGGGGGGGC
T. devil        508 GCTGGTTCCGGGGCGCGCGGAGCCCCAAGCTCGGGGAGGC
Wombat          167 GCTCGTTCCGGGGCGCGCGGAGCCCGAAGCCCGGGTGGGC
Consensus           GC CGTTC GGG CGC CGGAGCCCGAAGCCCGGGGGGGC

Opossum         552 GGGGGCGAAGCCTCCGCTCTGTCTCCTGCTGCGCCTGGGG
Koala           521 GGGGGCGCAGCCTCGGCTCTGCCTCCTGCTGAGCCTGGGG
T. devil        548 GGGGGCGCAGCCTCGGCTCTGTCTCCTCCTGCGCCTGGGG
Wombat          207 TGGGGCGCAGCCTCGGCTCTGCCTCCTGCTGCGCCTGGGG
Consensus           GGGGGCG AGCCTC GCTCTGtCTCCTGCTGCGCCTGGGG

Opossum         592 CTGCTATTGCTCCGCTTCAGCCAGATTGCTGTTGCA
Koala           561 TTGCTACTGCTCCGCTTCGGCCAGATTGCAGTTGCA
T. devil        588 CTGCTCCTGCTCGCCTTCTGCCAGATTGCTGTTGCA
Wombat          247 CTGCTACTGCTCGGCTTCGGCCAGATTGCAGTTGCA
```

```
Consensus            CTGCTA TGCTCcGCTTC GCCAGATTGCtGTTGCA
```

The AUG/ATG codons (green), the regions encoding the conserved Arg-cluster (yellow) and the hydrophobic region (grey) are marked. The signal peptidase cleavage sites were predicted *in silico*.
