# Supplementary material for: The translation attenuating arginine-rich sequence in the extended signal peptide of the protein-tyrosine phosphatase PTPRJ/DEP1 is conserved in mammals
Source: PLoS One. 2020 Dec 9;15(12):e0240498. doi: 10.1371/journal.pone.0240498 (PMC7725344; doi:10.1371/journal.pone.0240498)
Supplement: S4 Fig — (PDF) [file pone.0240498.s004.pdf]

S4 Fig. Alignment of the extended signal peptides of PTPRJ in marsupials.

```
Opossum          1 MSPGKPGAGGAERRRRSWRRRRRRRPRPRPPAPA------
Koala            1 MSPGKPGAGGAERRRRSWRRRRRRRPRPRPPAPA------
T.devil          1 MSPGKPGAGGAEKRRRTWRRRRRRRPRPGPPAAAPGAQAA
Wombat           1 MSPGKPGAGGAERRRRSWRRRRRRRPRPRPPA--------
Consensus          MSPGKPGAGGAERRRRSWRRRRRRRPRPRPPApA

Opossum         35 AVPGAEAAA-EAAALGPQRAALLPGTFRDARSPKPGGAGA
Koala           35 --PGAEAAA-EAAAPGPRRAALLPGSFRGARSPKPGGAGA
T.devil         41 AAPGAQAAAAEAAAPGPRRAAPLPGWFRGARSPKLGEAGA
Wombat          33 AAPGAEAAA-EAAAPGPRRAALLPGSFRGARSPKPGWAGA
Consensus          A PGAEAAA EAAA GP RAALLPG FR ARSPKPGgAGA

Opossum         74 KPPLCLLLRLGLLLLRFSQIAVA▼VS
Koala           72 QPRLCLLLSLGLLLLRFGQIAVA▼GD
T.devil         81 QPRLCLLLRLGLLLLAFCQIAVA▼DD
Wombat          72 QPRLCLLLRLGLLLLGFGQIAVA▼GD
Consensus           P LCLLLRLGLLLLrF QIAVA
```

The initiating Met residues (green), the conserved Arg-clusters (yellow), the hydrophobic regions (grey), and the cleavage sites of the signal peptidase (▼) are shown.
