# Supplementary material for: The translation attenuating arginine-rich sequence in the extended signal peptide of the protein-tyrosine phosphatase PTPRJ/DEP1 is conserved in mammals
Source: PLoS One. 2020 Dec 9;15(12):e0240498. doi: 10.1371/journal.pone.0240498 (PMC7725344; doi:10.1371/journal.pone.0240498)
Supplement: S5 Fig — (PDF) [file pone.0240498.s005.pdf]

S5 Fig. The 5′ end region of the *PTPRJ* mRNA in platypus encoding the extended signal peptide.

```
  1  GGCTGCAAAA GCAGCAGGAG CTGCAGTTGC AGCAATCGCA GCAGCAGCAG
 51  CAATTGCAAC AGCAGTTGCA GCAGCAGCAG CAATTGCAAC CTCCGCAGCG
101  GCAATTGCAG CAGCAACGGC CGGGCCGCGG TCCCTCCCCC CCGCGCCAAC
151  CCTGCCCGTT GCGGCCGGCC GAGGGCTGCT GCGGGGCCGT TGCGGCCGGA
201  GGCGCGCCCG CCCCCCCGCC ATGTCCCCGG GGAAGCCCGG AGCGGGGGAG
251  ACGCCTCCGA GGAGGAGGAG GCGGCGGGGG AGGCGGAGGA GGAGGAGGAG
301  GAGGCCCCAG CCGGGACCGG CGACGACGAA GGCGGCGGCG GGTGGAGCCG
351  GGCCCCGGCT TGCGGGCCTC CCGGGAAGGC TGGGCGGCAT GAAGCTCGGC
401  TCCCTGCTGG GGCTGCTCTT GCTGCTGCAT TCCGGACAGA TGAGATGT
```

The in-frame AUG codons upstream of the hydrophobic region (green), the region encoding the Arg-cluster (yellow) and the hydrophobic region (grey) are marked. The cleavage site of the signal peptide was predicted *in silico*.
