# Supplementary material for: The translation attenuating arginine-rich sequence in the extended signal peptide of the protein-tyrosine phosphatase PTPRJ/DEP1 is conserved in mammals
Source: PLoS One. 2020 Dec 9;15(12):e0240498. doi: 10.1371/journal.pone.0240498 (PMC7725344; doi:10.1371/journal.pone.0240498)
Supplement: S6 Fig — (PDF) [file pone.0240498.s006.pdf]

S6 Fig. The extended signal peptide of PTPRJ in platypus.

```
 1   MSPGKPGAGE TPPRRRRRRG RRRRRRRRPQ PGPATTKAAA GGAGPRLAGL
51   PGRLGGMKLG SLLGLLLLLH SGQMRC▼AG
```

The initiating Met residue (green), the Arg-cluster (yellow), the hydrophobic region (grey), and the signal peptidase cleavage site (▼) are shown.
