# Supplementary material for: The translation attenuating arginine-rich sequence in the extended signal peptide of the protein-tyrosine phosphatase PTPRJ/DEP1 is conserved in mammals
Source: PLoS One. 2020 Dec 9;15(12):e0240498. doi: 10.1371/journal.pone.0240498 (PMC7725344; doi:10.1371/journal.pone.0240498)
Supplement: Attachment — Submitted filename: Response to reviewers_101120.docx [file pone.0240498.s007.docx]

| **Maria Gasset, Ph.d.**  **Academic Editor**  **PLOS ONE** | **Prof. Dr. Frank-D.Böhmer**  (HD i.R.)  Institute of Molecular Cell Biology  Hans-Knöll-Strasse 2  07745 Jena  Phone: +49 36427 71381  E-Mail: i5frbo@uni-jena.de |
| --- | --- |

November 10, 2020

Dear Dr. Gasset,

Many thanks for the review of our manuscript and the possibility to revise it. We are very grateful for all of the remarks and suggestions of the reviewer and made the appropriate amendments and corrections to the manuscript. Amendments in response to the review are labeled red in the manuscript with highlighted changes.

Our point-by-point response to the comments of Reviewer #1 is below:

*When I look up human PTPRJ in UniProt (Q12913), I only see the "short" sequence, translated from the third AUG, with a predicted signal peptide of 35 amino acids. The authors should report their findings to UniProt.*

Thank you very much for this suggestion! We have submitted a request to UniProt for amendment of the Q12913 entry quoting our earlier work and the current manuscript. We hope very much that an appropriate alteration of the entry will be conducted in due time.

Major comments:

*1 - p.1 (and several other places in the manuscript): "... an N-terminal extension and a recessed hydrophobic signal sequence (Hiss et al., 2008)."; two comments:*

** It is not clear what the authors mean by "recessed" - does it just mean that the hydrophobic region is far from the N-terminus? Hiss et al. do not use this term. Please define.*

We have now avoided throughout the manuscript using the descriptive term “recessed” and instead simply indicate that the hydrophobic region is distant/far away from the N-terminus.

** It is not customary to refer to the hydrophobic part of a signal peptide as "signal sequence"; instead, it is normally called "h-region". This is also how Hiss et al. refer to it, while "Signal sequence" in Hiss et al. is used as a synonym for signal peptide. Please use "h-region" instead throughout the manuscript.*

We have altered the manuscript accordingly and use now throughout in text and figures the term “hydrophobic region” or “h-region” instead of “signal sequence”.

*2 - p.2: "The NCBI gene database was used to search for PTPRJ orthologs in mammals": More detail is needed here. There are various ways of operationally defining orthology, so the authors should precisely describe how this was done.*

We describe now in more detail the procedure of finding human *PTPRJ* orthologs, that is genes in other organisms, which are similar to human *PTPRJ* due to common descent. The related text (p.4) spells now: ‘*The NCBI gene database (https://www.ncbi.nlm.nih.gov/gene) entry for the human protein tyrosine phosphatase receptor type J (PTPRJ, GeneID: 5795) was used to search for PTPRJ orthologs. The NCBI default routine designated ‘NCBI's Eukaryotic Genome Annotation pipeline’ was used, which employs the NCBI Gene dataset and a combination of protein sequence similarity and local synteny information (https://www.ncbi.nlm.nih.gov/kis/info/how-are-orthologs-calculated/). The search for orthologs was limited to mammals.*’

*3 - p.4: "The N-termini of the proteins targeted for secretion or membrane integration, harbor a short amino acid sequence – the signal peptide...": regarding proteins targeted for membrane integration, this is only true for some of them.*

We modified the sentence (now at p.11) to indicate that not all proteins targeted for membrane integration contain a signal peptide.

*4 - p.5: "Recent experiments showed that the N-terminal extension of the signal peptides is irrelevant for the signal sequence function (Meyer et al., 2018)": it should be mentioned that Meyer et al's results did not concern mammals.*

We indicated that this observation was made in *Plasmodium falciparum* (p.11).

*5 - Just a suggestion: I submitted the human nucleotide sequence (NM_002843.4) to the NetStart web server for prediction of start codons (https://services.healthtech.dtu.dk/service.php?NetStart-1.0), and indeed, AUG-190 scores higher than AUG-355 and AUG-13. Maybe the authors would like to do this for all their nucleotide sequences and see if this is a general observation?*

We followed this very valuable suggestion. The different AUG codons to initiate translation *PTPRJ* nucleotide sequences from placental mammals and platypus were submitted to the NetStart-1.0 web server. Indeed, the AUG positioned prior to the Arg-cluster has in all cases the highest score and presumably is most active in initiating translation. This is included in the text (p.9-10).

Minor comments:

We corrected all mistakes accordingly.

Further changes:

Throughout we have adjusted the style of the manuscript to PLOS ONE standards. Notably, we have adjusted formatting of the manuscript text and of references, placed figure captions and Table 1 into the main text, adjusted features of the figure files, and placed captions for the supplementary figures at the end of the manuscript. These amendments are labeled blue in the manuscript with highlighted changes.

Yours sincerely
